# Supplementary material for: Facet-engineered TiO2 drives photocatalytic activity and stability of supported noble metal clusters during H2 evolution
Source: Nat Commun. 2023 Oct 3;14:6165. doi: 10.1038/s41467-023-41976-2 (PMC10547715; doi:10.1038/s41467-023-41976-2)
Supplement: Supplementary file 1 — Supplementary Information [file 41467_2023_41976_MOESM1_ESM.pdf]

Supplementary Information for

Facet-engineered TiO_2 drives photocatalytic activity and stability of supported noble metal clusters during H_2 evolution

Yufen Chen [1,2], Lluís Soler [1,2,*], Claudio Cazorla [3], Jana Oliveras [4], Neus G. Bastús [4], Víctor F. Puntes [4,5,6], Jordi Llorca [1,2,*]

[1] *Department of Chemical Engineering, Universitat Politècnica de Catalunya. Eduard Maristany 16, EEBE, Barcelona 08019, Spain*

[2] *Institute of Energy Technologies and Barcelona Research Center in Multiscale Science and Engineering, Universitat Politècnica de Catalunya. Eduard Maristany 16, EEBE, Barcelona 08019, Spain*

[3] *Department of Physics, Universitat Politècnica de Catalunya. Campus Nord, B4-B5, Barcelona E-08034, Spain*

[4] *Institut Català de Nanociència i Nanotecnologia (ICN2), CSIC and The Barcelona Institute of Science and Technology (BIST). Campus UAB, 08193, Barcelona, Spain*

[5] *Institució Catalana de Recerca I Estudis Avançats (ICREA). 08010, Barcelona, Spain*

[6] *Vall d'Hebron Research Institute (VHIR). Hospital Universitari Vall d'Hebron, Passeig de la Vall d'Hebron, 129, 08035 Barcelona, Spain.*

* corresponding authors: lluis.soler.turu@upc.edu, jordi.llorca@upc.edu

Supplementary Methods

Reagents

Gold (III) acetate (Alfa Aesar, 99.9%), Palladium (II) acetate (Acros Organics, 47.5% Pd), Platinum (II) acetylacetonate (Acros Organics, 98%) were used as metal precursors. Titanium (IV) fluoride (TiF_4, 99%), titanium (IV) chloride ($TiCl_4$, 99%), 1-octadecene (90%) (1-ODE), 1-octadecanol (1-ODOL, 97%). Oleylamine (OLAM, 70%) and oleic acid (OLAC, 90%) were obtained from Sigma-Aldrich. Absolute ethanol was obtained from Scharlau. Milli-Q water was routinely used.

Photocatalysts Preparation

The preparation of TiO_2 photocatalysts

TiO_2 nanoshapes exhibiting (001) and (101) facets were prepared following a surfactant-assisted synthesis reported by Gordon et al[1]. Highly monodisperse anatase tetragonal bipyramidal TiO_2 nanocrystals (NCs) exposing primarily {101} facets were produced using a mixture of TiF_4/$TiCl_4$ precursors in the presence of OLAM as a co-surfactant. Similarly, the use of 1-ODOL as a co-surfactant leads to TiO_2 nanoplates with a high percentage of {001} facets. TiO_2 NCs were prepared as follows: 30 mmol of cosurfactant (OLAM or 1-ODOL), 10.2 mL of 1-ODE, and 0.48 mL (1.5 mmol) of OLAC were degassed at 120 °C for 1 h. After, 1.5 mL of a mixed TiF_4/$TiCl_4$ stock solution 1:1 was added at 60 °C and the solution was quickly heated to 290 °C. After 10 min, 8 mL of the stock solution was pumped into the flask at 0.3 mL min^{-1} using a syringe pump. Afterwards, the heating mantle was removed, and the flask was left to cool naturally to ambient temperature. After the synthesis, a mixture of 2-propanol and methanol is added to precipitate the NCs. Centrifugation at 4951 × g for 10 min is used to recover them. This washing process was repeated twice. For the mixture of TiF_4/$TiCl_4$ precursors, TiF_4 and $TiCl_4$ stock solutions were mixed at equal volume in the glovebox. TiF_4 stock solution consists of 0.2 M TiF_4 and 1.0 M OLAC in 1-ODE. $TiCl_4$ stock solution consists of 0.2 M $TiCl_4$ and 1.0 M OLAC in 1-ODE. The TiF_4 stock solution is stirred on a hot plate set to 80 °C to promote the dissolution of TiF_4. Once dissolved, the TiF_4 stock solution is orange-brown, and the $TiCl_4$ stock solution is dark brown.

The TiO_2 anatase suspensions were evaporated overnight at room temperature. The obtained anatase powders were analysed through thermogravimetric measurements (TGA) to assess the temperature of complete mass loss of residual surfactants on the surface (Supplementary Fig.

19). Both materials were calcined in air at 500 °C for 1 h (2 °C min^{-1}) and the resulting materials were labelled as TiO_2-001 (for plates exhibiting {001} facets) and TiO_2-101 (for bipyramids exhibiting {101} facets). To facilitate an intuitive nomenclature of the samples that reflects the information of the primarily titania facet exposed, we named the obtained samples as TiO_2-{hkl}. The XRD patterns (Supplementary Fig. 3) was used to confirm the formation of crystal facets of anatase without any impurities or rutile phase. TEM images (Supplementary Fig. 1a and 1b) unambiguously showed the synthesized the shapes of anatase plates and bipyramids. At least one hundred TiO_2 nanoparticles in each sample were considered for estimating the particle size/shape distribution (Supplementary Fig. 2). On the basis of the obtained structural information, the percentages of preferentially exposed (101) facets in TiO_2 bipyramids and (001) facets in TiO_2 plates were estimated to be 91%–97% and 50%–76%, respectively. The detailed calculations are shown in Supplementary Fig. 20. The Fourier Transform (FT) images of fresh Pd/TiO_2 provide direct evidence of crystal planes of anatase {001} facets and {101} facets (Supplementary Figs. 1c and 1d, respectively).

The preparation of M-TiO_2 photocatalysts

The mechanochemical preparation of the photocatalysts was performed in a high-energy RETSCH Mixer Mill MM200, using a 10 mL stainless-steel vial and one stainless-steel milling ball (d = 15 mm, m = 13.54 g), as described elsewhere[2,3]. Briefly, 0.0057 g of gold acetate, 0.0063 g of palladium acetate or 0.0061 g of platinum acetylacetonate was mixed with 0.2943 g, 0.2937 g or 0.2939 g of TiO_2, respectively, leading to a ball to powder weight ratio (BPR) of 45 and metal loading of 1 wt%. Milling was performed at 15 Hz for 10 min. The resulting metal/TiO_2 samples were denoted as M-TiO_2-{hkl}, which are Au/TiO_2-001, Au/TiO_2-101, Pd/TiO_2-001, Pd/TiO_2-101, Pt/TiO_2-001 and Pt/TiO_2-101. The obtained photocatalysts were used for the photocatalytic experiments without any further treatment. The actual loading amount of Au, Pd and Pt was quantified by Inductively Coupled Plasma-Optical Emission Spectrometry (ICP-OES, Perkin Elmer Optima 3200RL).

Photocatalysts Characterization

X-ray diffraction (XRD) data were collected on a PANalytical X'Pert diffractometer using a Cu Kα radiation source (λ = 1.541 Å). In a typical experiment, the 2θ diffraction (Bragg) angles were measured by scanning the goniometer from 10° to 100°. The samples were prepared by centrifugation to precipitate the NCs. The supernatant was discarded, and samples were dried at room temperature. Peak positions were determined using the X'Pert HighScore program

after baseline correction. High-angle annular dark field scanning transmission electron microscopy (HAADF-STEM) and high-resolution transmission electron microscopy (HRTEM) images were acquired on a FEI Tecnai G2 F20 instrument equipped with a field emission gun operating at an accelerating voltage of 200 kV. At least one hundred of Au, Pd and Pt particles in each photocatalyst were collected to estimate the size distribution. X-ray photoelectron spectroscopy (XPS) was conducted on a SPECS system. The apparatus was equipped with an XR-50 X-ray source (Al anode at 150 W), a PHOIBOS 150 EP hemispherical energy analyser and an MCD-9 detector. The spectra were corrected with the position of the adventitious carbon 1s signal at 284.8 eV. Thermogravimetric analyses (TGA) were performed on the TA Instrument with a Q50 system under nitrogen atmosphere (10 °C/min). The actual loading amount of Au, Pd and Pt was quantified by Inductively Coupled Plasma-Optical Emission Spectrometry (ICP-OES, Perkin Elmer Optima 3200RL). Surface area measurements were conducted on an automatic Micromeritics ASAP 2020 analyzer using N_2 adsorption isotherms and BET (Brunauer−Emmett−Teller) surface area analysis methods. Samples were degassed under vacuum at 200 °C for 4 h before adsorption measurements. The average pore diameter distributions were derived from the desorption branches of the isotherms based on Barrett-Joyner-Halenda (BJH) model.

Photocatalytic Hydrogen Production

The photocatalytic hydrogen evolution reaction was conducted in a 40 mL tubular glass photoreactor at room temperature and atmospheric pressure under dynamic conditions. An UV lamp equipped with four LEDs emitting at 365 ± 5 nm (SACOPA, S.A.U.) was employed as the light source. An argon gas (Ar) stream (20 mL min^{-1}) was passed through a saturator (Dreschel bottle), which contained a liquid mixture of water (H_2O, 150 g) and ethanol (EtOH, 17 g). The resulting gaseous mixture of H_2O, EtOH and Ar was directly introduced into the photoreactor. 2.0 mg of each photocatalyst was dispersed in 0.5 mL absolute ethanol and treated by ultrasonication for 10 min to form a homogeneous suspension. Afterwards, the slurry was dropped onto a circular cellulose paper (from Albet, thickness 0.18 mm, area 2.54 cm^2) and dried at 50 °C for 1 h. The impregnated paper was placed upside down in the middle of the glass photoreactor with two separated tubular sections, along with an O-ring (Supplementary Fig. 6). The light source was positioned at the bottom of the photoreactor. By aligning a synthetic quartz glass cylindrical lens from the light source to the cellulose paper loaded with photocatalyst, 80.5 ± 0.5 mW cm^{-2} of UV irradiance reached the sample. The temperature of the photocatalyst under the UV irradiation was monitored directly with a K-type thermocouple

in contact with the sample. The gas hourly space velocity (GHSV) was 26000 h^{-1} and the contact time was 0.14 s. Prior to the photoreaction, the system was purged with Ar gas (20 mL min^{-1}) for 30 min. The products evolved from the outlet of photoreactor (hydrogen and acetaldehyde in equimolar amount, $C_2H_5OH \rightarrow H_2 + CH_3CHO$) were analysed on-line every 4 min with a micro-gas chromatograph (GC, Agilent 490)[4,5].

First-Principles Simulation Techniques

We performed spin-polarized first-principles calculations based on density functional theory (DFT) for anatase TiO_2 surfaces functionalised with Au, Pd and Pt co-catalysts. The PBE functional[6] was used as implemented in the VASP software package[7]. A "Hubbard-*U*" scheme[8] with $U = 3$ eV was employed for a better treatment of the localized Ti *d* electronic orbitals (Hubbard-like corrections were not applied on the noble metal atoms). The value of the lattice parameters, however, were constrained to their corresponding experimental values of $a_0 = b_0 =$ 3.78 Å and $c_0 = 9.51$ Å[9] since these are not correctly reproduced by the PBE+*U* approach (see the last two paragraphs in this section and Supplementary Fig. 21). We used the "projector augmented wave" method to represent the ionic cores[10] by considering the following electrons as valence: Au 5*d*, and 6*s*; Pd 4*d*; Pt 5*d* and 6*s*; Ti 3*d*, 4*s*, and 3*p*; and O 2*s* and 2*p*. Wave functions were represented in a plane-wave basis truncated at 650 eV. For integrations within the first Brillouin zone, a Monkhorst-Pack k-point grid was employed with a density equivalent to 16x16x12 for the anatase unit cell. Geometry relaxations were performed by using a conjugate-gradient algorithm that allowed for cell shape variations; the geometry relaxations were halted when the forces on the atoms fell all below 0.005 eV$Å^{-1}$. By using these technical parameters, we obtained zero-temperature energies converged to within 0.5 meV per formula unit.

All the DFT geometry relaxations in the present work were performed at the PBE+*U* level with $U = 3$ eV and by constraining the size of the lattice vectors to their experimental values; the positions of the atoms, however, were allowed to fully relax in all the cases. Subsequently, the energy, charge density, and optoelectronic properties of anatase TiO_2 were estimated with the range-separated hybrid HSE06 exchange-correlation functional without performing further relaxations (see the last two paragraphs in this section and Supplementary Fig. 21).

The slab supercells were constructed as a 4x4x4 repetition of the anatase unit cell and a vacuum region of 25 Å thickness was considered in all the simulations. Adsorption energies were calculated with the formula $E_{ads} = E_{M@anatase} - E_{anatase} - E_{M}$, where $E_{M@anatase}$ represents the

energy of the blended noble metal-TiO_2 system, $E_{anatase}$ the energy of the anatase slab, and E_M the energy of the isolated noble metal atom or cluster. To estimate accurate energy band gaps, density of electronic states and charge redistributions at affordable computational expense, we employed the hybrid HSE06 exchange-correlation functional[11] and adopted the equilibrium geometries determined at the PBE+U level. In order to simulate photoexcitation effects in TiO_2-based photocatalysts, we employed an effective DFT approach, equivalent to those employed in previous works[12,13], that consists in constraining the partial occupancies of each electronic orbital by adjusting the width of the corresponding Fermi smearing. In particular, a Fermi smearing of σ (eV) in the constrained DFT calculations implies considering the electronic occupation function $f(E,\sigma) = 1/(\exp[(E-E_F)/\sigma]+1)$ instead of the usual step function $f(E) = 1$ for $E \leq E_F$ and $f(E) = 0$ for $E > E_F$, where E_F represents the Fermi energy level.

Several tests were undertaken for assessing the performance of the PBE+U approach in predicting the lattice parameters and optoelectronic properties of bulk anatase TiO_2 (Supplementary Fig. 21). In particular, we found that for the PBE+U approach to render a theoretical band gap close to the experimental value of 3.2 eV one needs to increase the size of the U parameter beyond a reasonable value of 9 eV (Supplementary Fig. 21b). Essentially, the PBE+U approach tends to underestimate the energy difference between the bottom of the conduction band and the Fermi energy level (Supplementary Fig. 21d). However, upon increasing the value of U the obtained relaxed lattice parameters increasingly depart from the experimental a_0 and c_0 values (Supplementary Fig. 21a). Similar test calculations were performed for the PBEsol+U exchange-correlation functional (not shown here), arriving at the same conclusions.

The reason for adopting the present combined PBE+U/HSE06 computational approach is twofold. First, full geometry relaxations of the slabs simulated in this study are not computationally feasible with the hybrid HSE06 exchange-correlation functional. And second, in the Supplementary Fig. 21c it is clearly appreciated that practically any anatase geometry generated at the PBE+U level, by constraining the lattice vectors to their experimental values, renders a HSE06 band gap that is in excellent agreement with the experimental value of 3.2 eV. Therefore, it can be concluded that the adopted computational approach is very robust because (1) it reproduces the structural and optoelectronic properties of anatase TiO_2 as they have been measured in experiments, and (2) the specific value of U that is adopted for the calculations has a very minor impact on the estimated optoelectronic properties.

Supplementary Figures

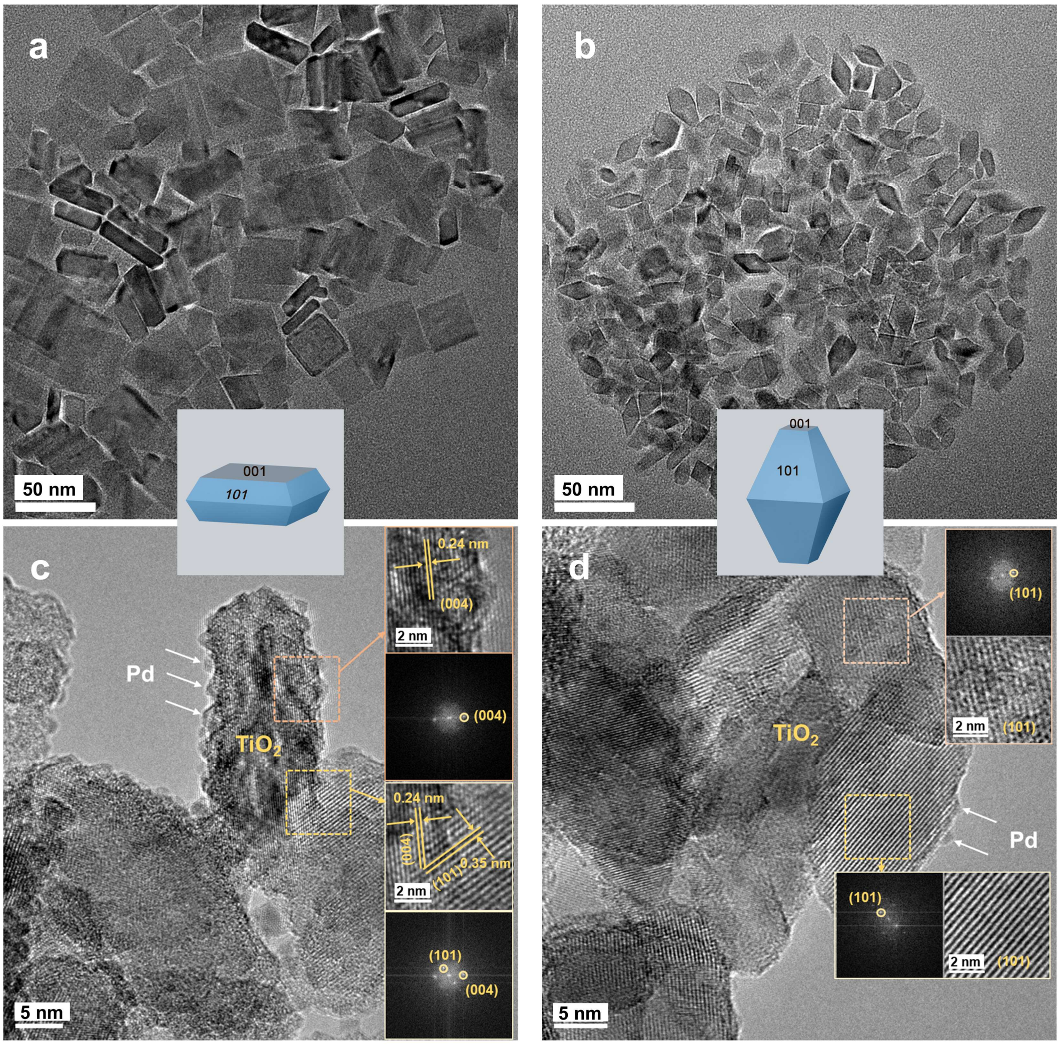

Supplementary Figure 1. Bright-field TEM images and Fourier Transform (FT) images corresponding to the crystal planes of (a, c), TiO_2 plates with preferentially exposed {001} facets and (b, d), TiO_2 bipyramids with preferentially exposed {101} facets.

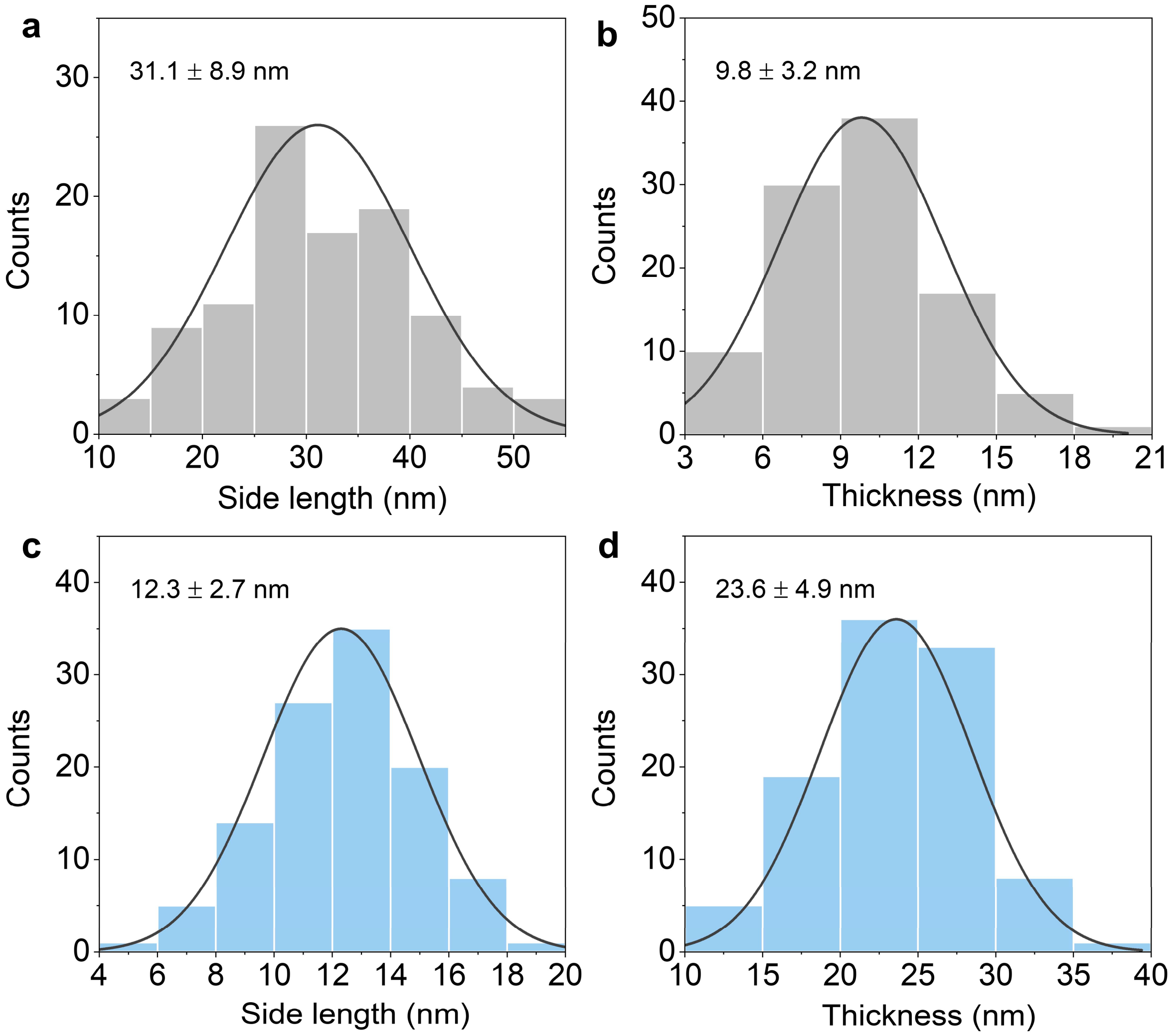

Supplementary Figure 2. Size distribution of TiO_2-001 (a,b, plates) and TiO_2-101 (c,d, bipyramids).

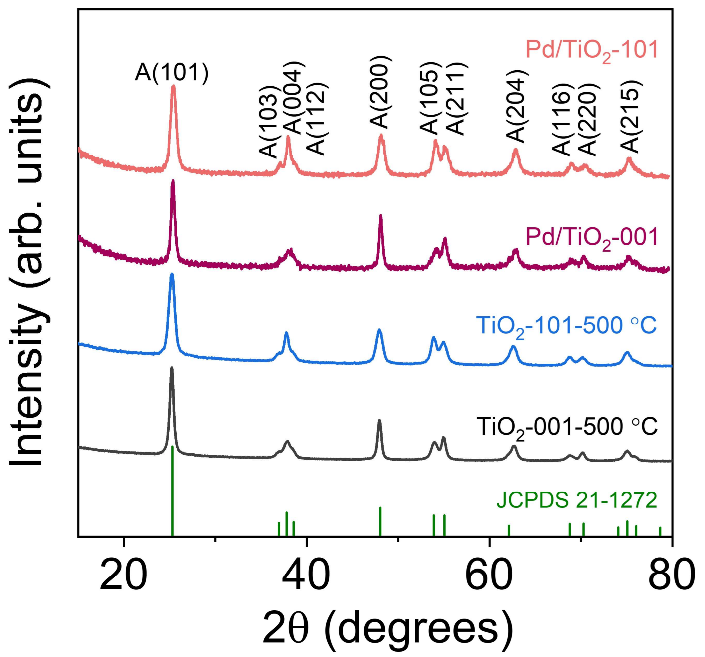

Supplementary Figure 3. XRD patterns of TiO_2-001 (plates) and TiO_2-101 (bipyramids) after calcination at 500 °C for 1 h. The XRD patterns of TiO_2 NCs show sharp and intense peaks corresponding to the crystal facets of anatase TiO_2 [JCPDS No. 021-1272 card] without any impurities of the remaining precursor used each time or the formation of another phase such as rutile. The XRD narrow peaks show an anisotropic growth of the NCs. In the case of bipyramids, this is indicated by the enhanced peak intensity and narrow width of the (004) reflection and relatively lower intensity and broader width for the other reflections. Obtained results are in agreement with those previously published for similar crystal structures[1].

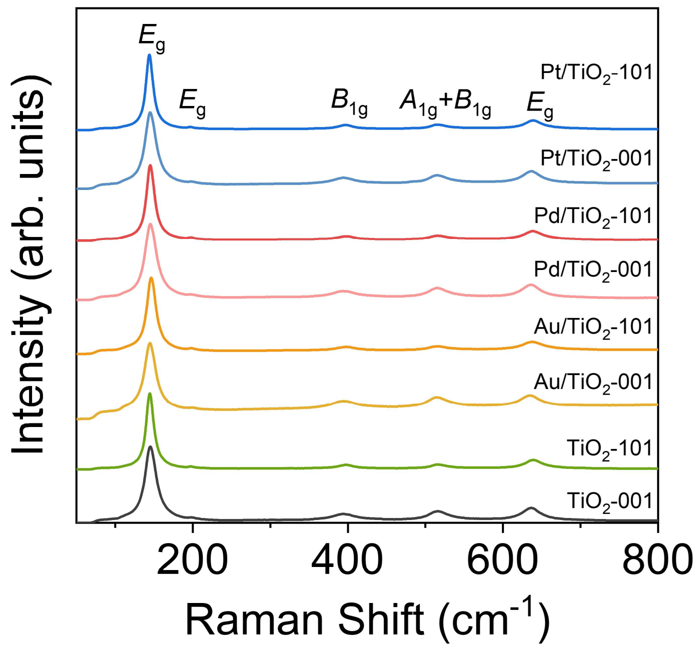

Supplementary Figure 4. Raman spectra of TiO_2-001, TiO_2-101, and M/TiO_2-{hkl} samples.

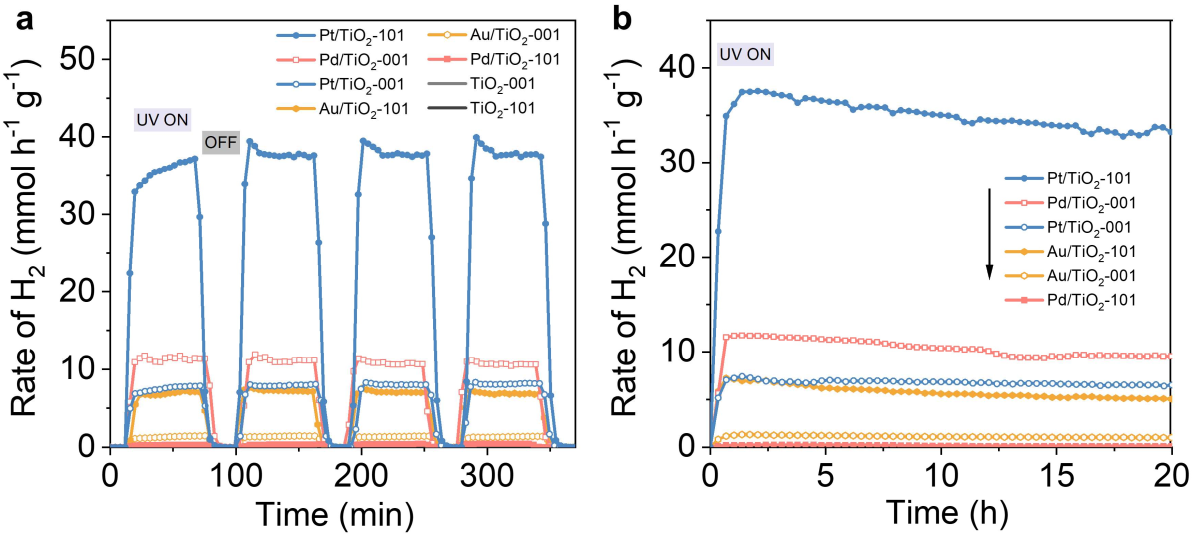

Supplementary Figure 5. 4 × 1 h light on-off cycle experiments (a) and 20-hour stability tests (b) of all as-prepared samples for photocatalytic H_2 production.

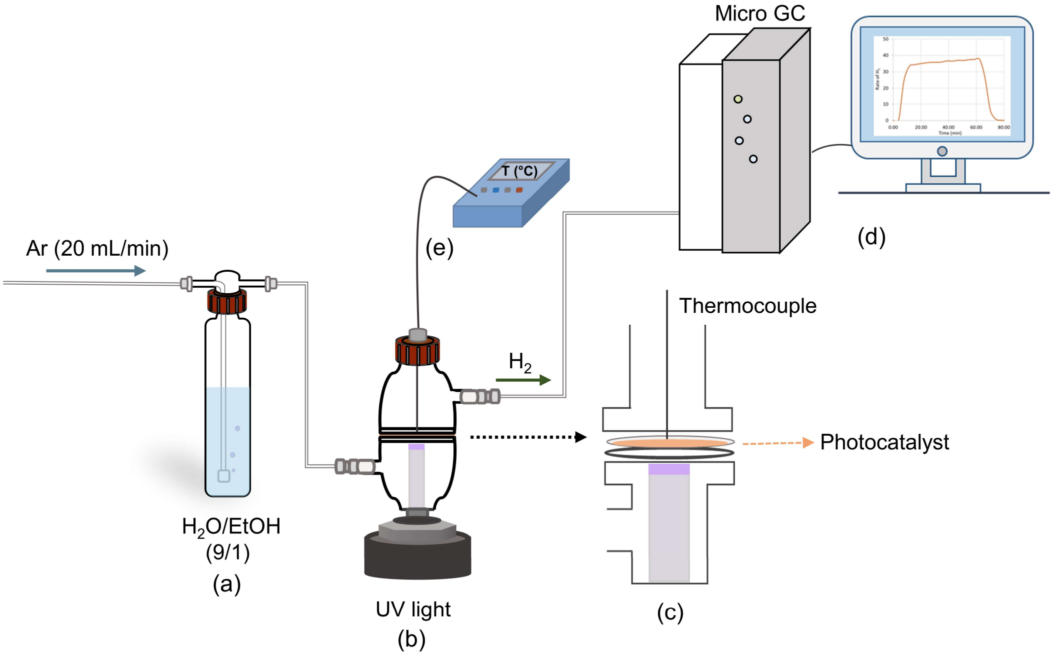

Supplementary Figure 6. Photocatalytic reactor for hydrogen production. (a): saturator containing the mixture of H_2O/EtOH (9/1). (b): photoreactor equipped with an UV lamp in the bottom. (c): enlarged scheme of (b). The impregnated paper was placed upside down in the middle of the glass photoreactor with two separated tubular sections, along with an O-ring. (d): micro-gas chromatograph and the monitor system. (e): K-type thermocouple.

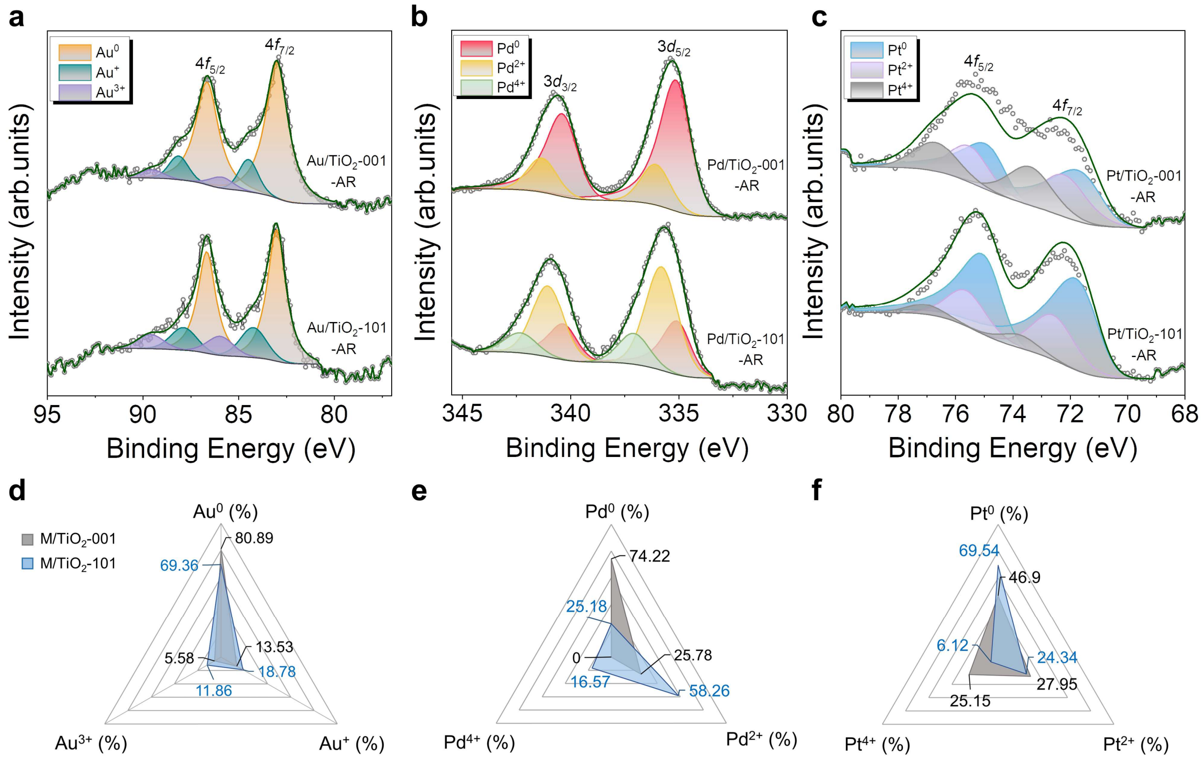

Supplementary Figure 7. XPS results of Au/TiO_2 and Pd/TiO_2, Pt/TiO_2 photocatalysts after 20 hours of photoreaction. XP Au 4*f* (a), Pd 3*d* (b) and Pt 4*f* (c) spectra of post-reacted Au/TiO_2-001, Au/TiO_2-101, Pd/TiO_2-001, Pd/TiO_2-101, Pt/TiO_2-001 and Pt/TiO_2-101 photocatalysts. The corresponding ratios of metal oxidation states extracted from XP spectra in (a), (b) and (c) are shown in (d), (e), and (f) respectively.

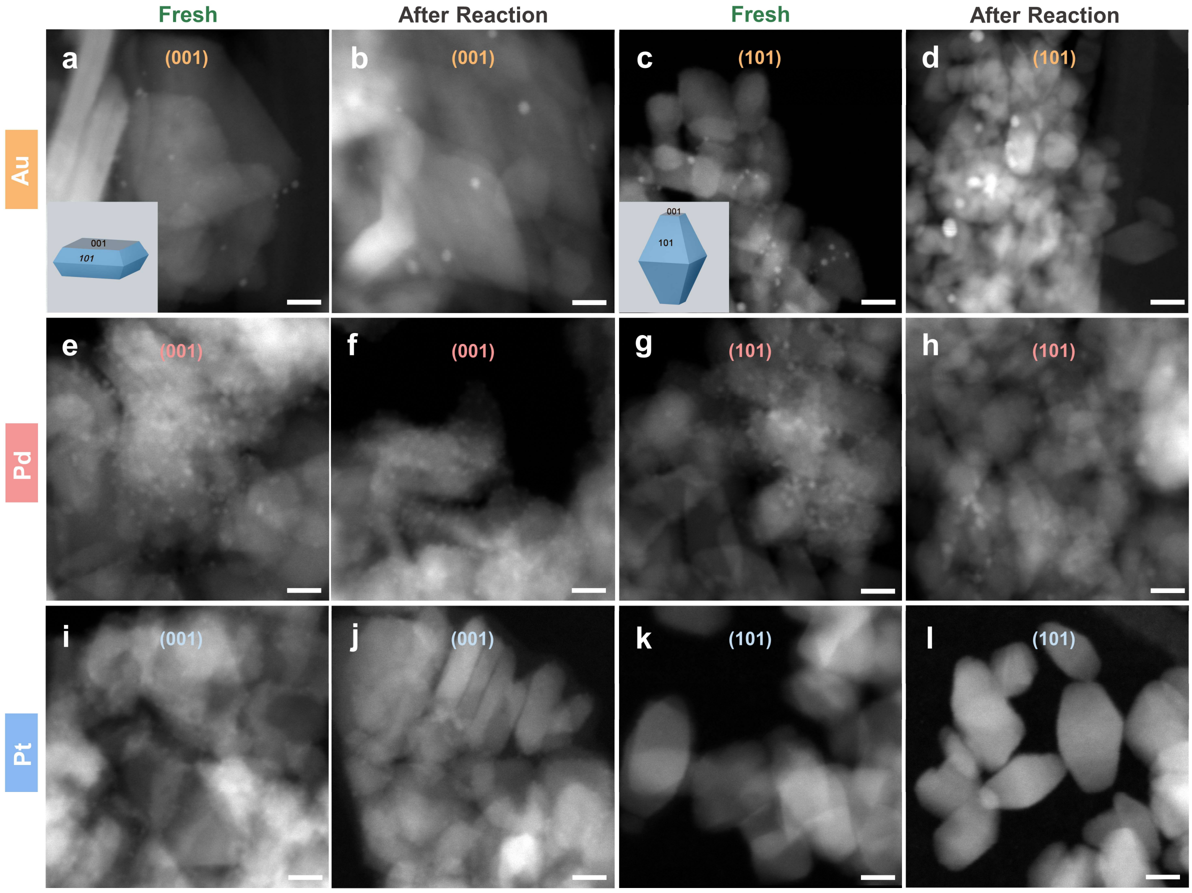

Supplementary Figure 8. HAADF-STEM images of fresh (a, c, e, g, i, k) and after reaction (b, d, f, h, j, l) samples. a,b, Au/TiO_2-001. c,d, Au/TiO_2-101. e,f, Pd/TiO_2-001. g,h, Pd/TiO_2-101. i,j, Pt/TiO_2-001. k,l, Pt/TiO_2-101. Scale bar: 10 nm. The bright dots refer to the corresponding metal clusters in each photocatalyst.

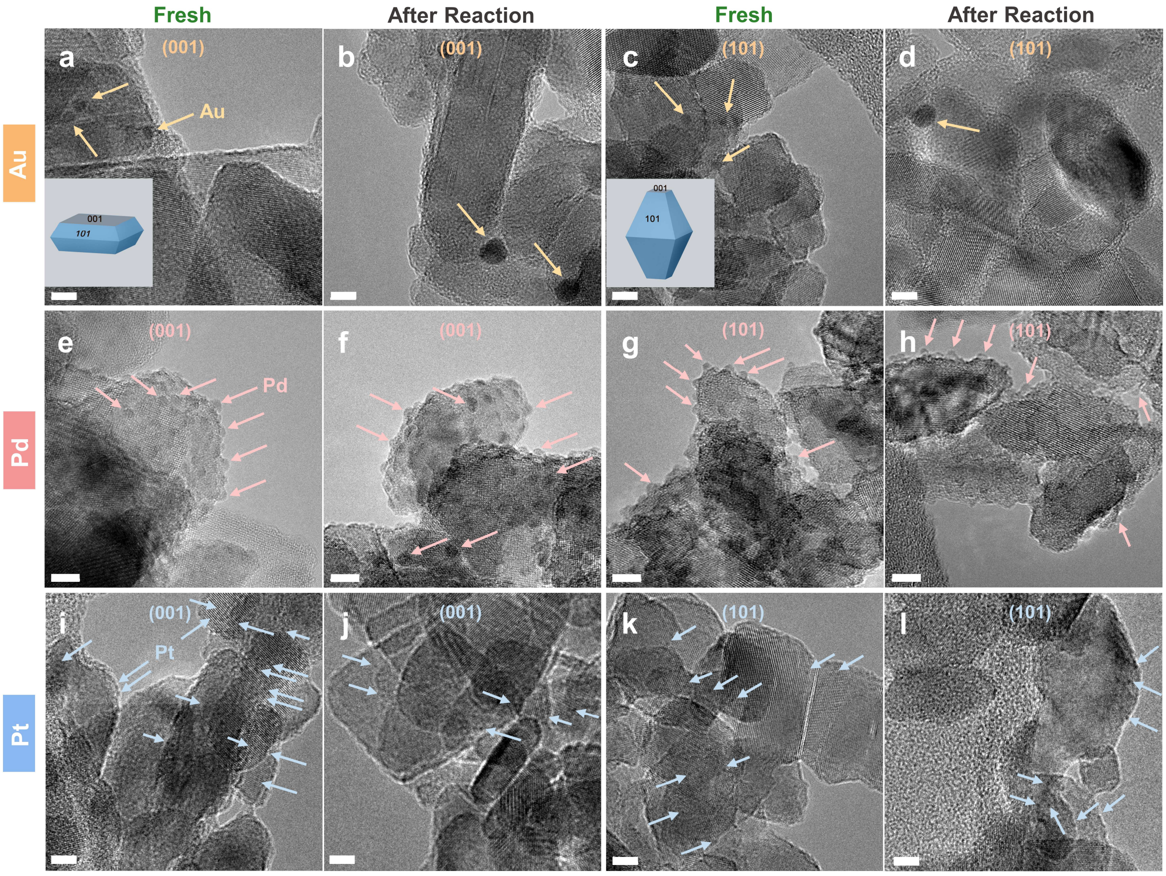

Supplementary Figure 9. HRTEM images of the metal clusters of the fresh and after photoreaction samples. a,b, Au/TiO_2-001. c,d, Au/TiO_2-101. e,f, Pd/TiO_2-001. g,h, Pd/TiO_2-101. i,j, Pt/TiO_2-001. k,l, Pt/TiO_2-101. Scale bar: 5 nm. Metal clusters are indicated by arrows.

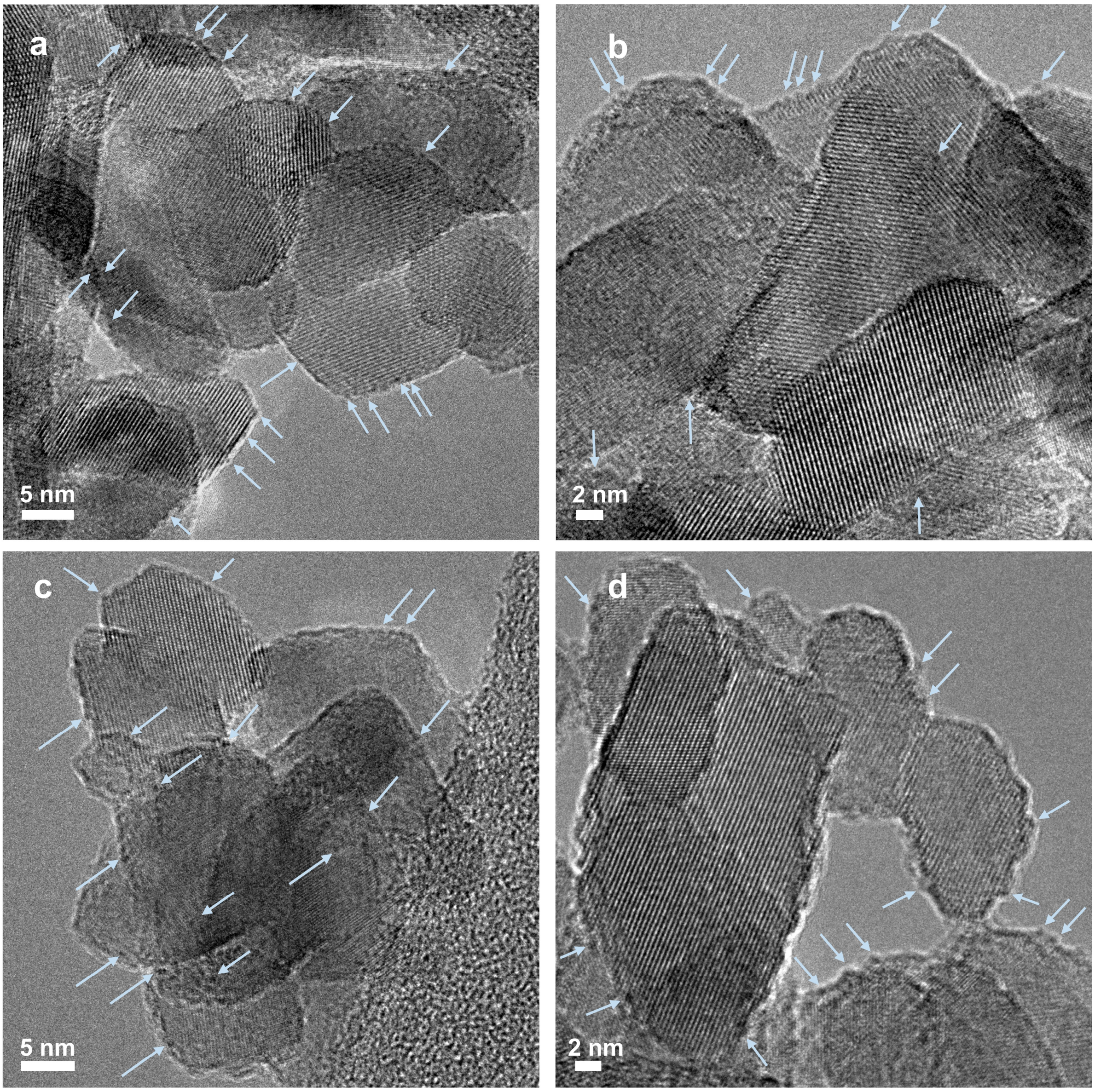

Supplementary Figure 10. HRTEM images of fresh Pt/TiO_2-001 (a,b) and Pt/TiO_2-101 (c,d) samples.

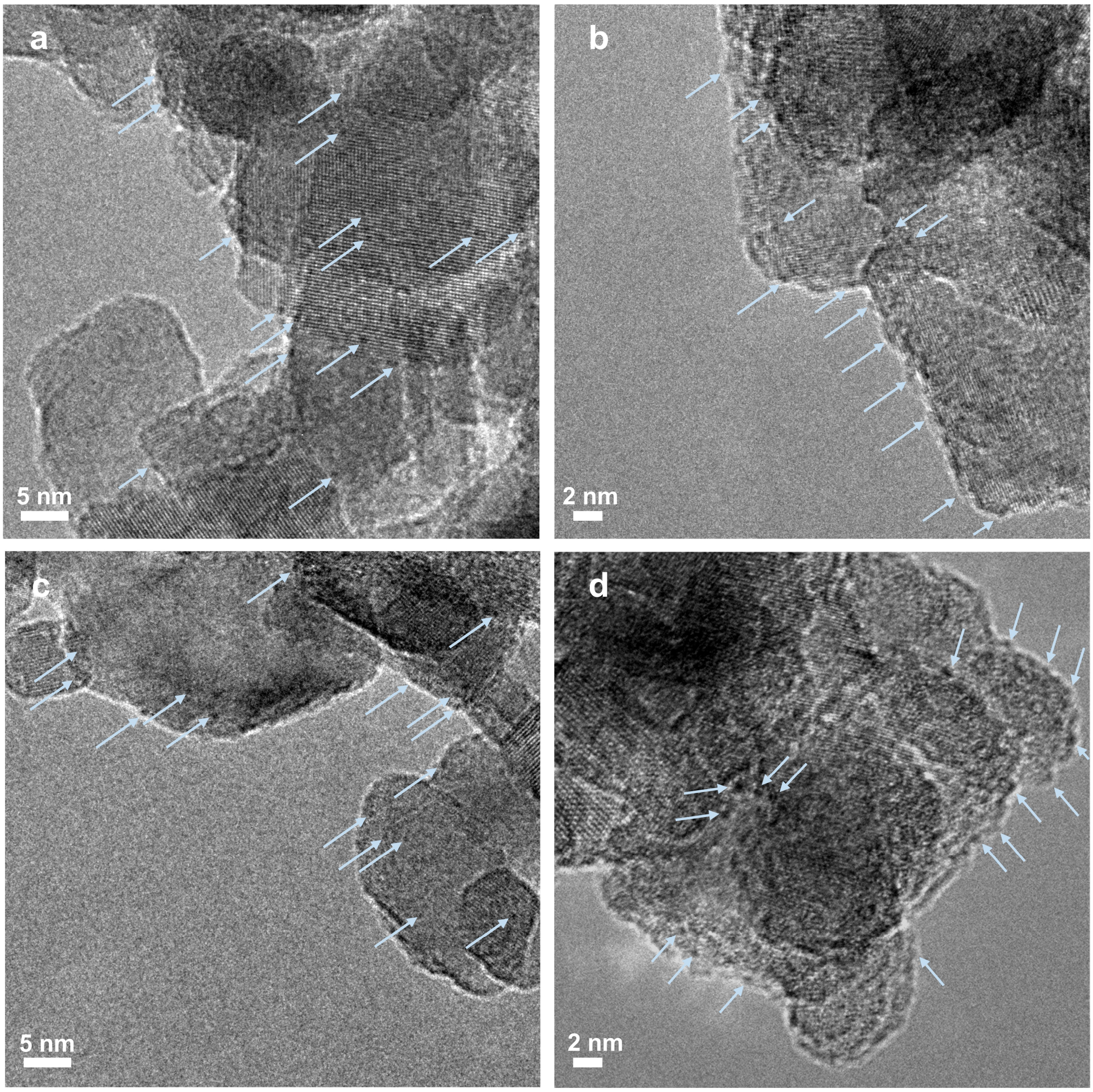

Supplementary Figure 11. HRTEM images of post-reacted Pt/TiO_2-001 (a,b) and Pt/TiO_2-101 (c,d) samples.

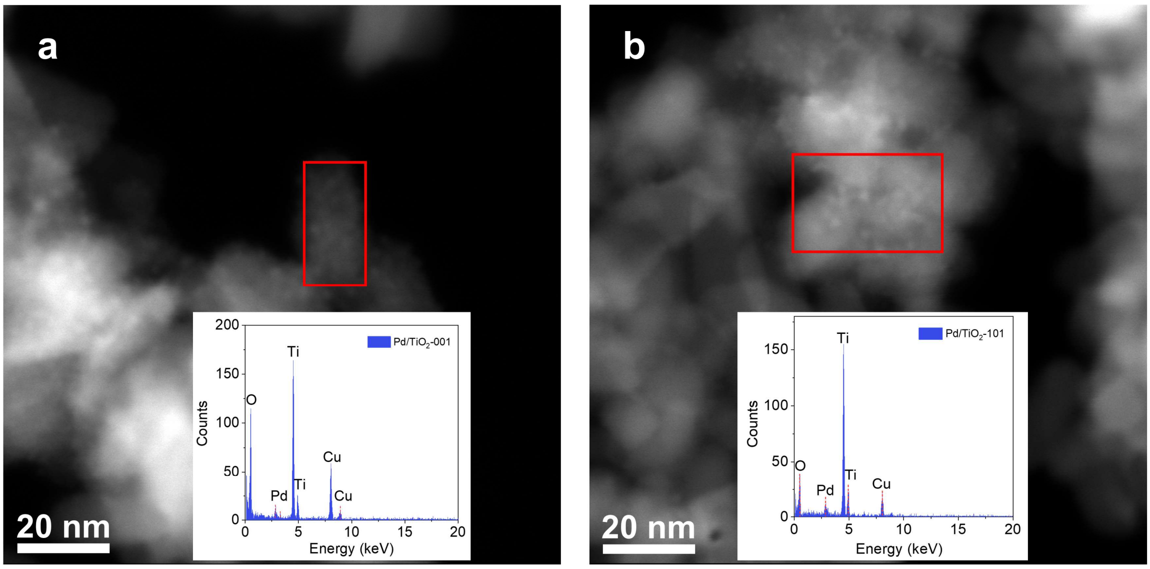

Supplementary Figure 12. HAADF-STEM images of fresh (a) Pd/TiO_2-001 and (b) Pd/TiO_2-101. EDX spectra were recorded in the marked area of each sample.

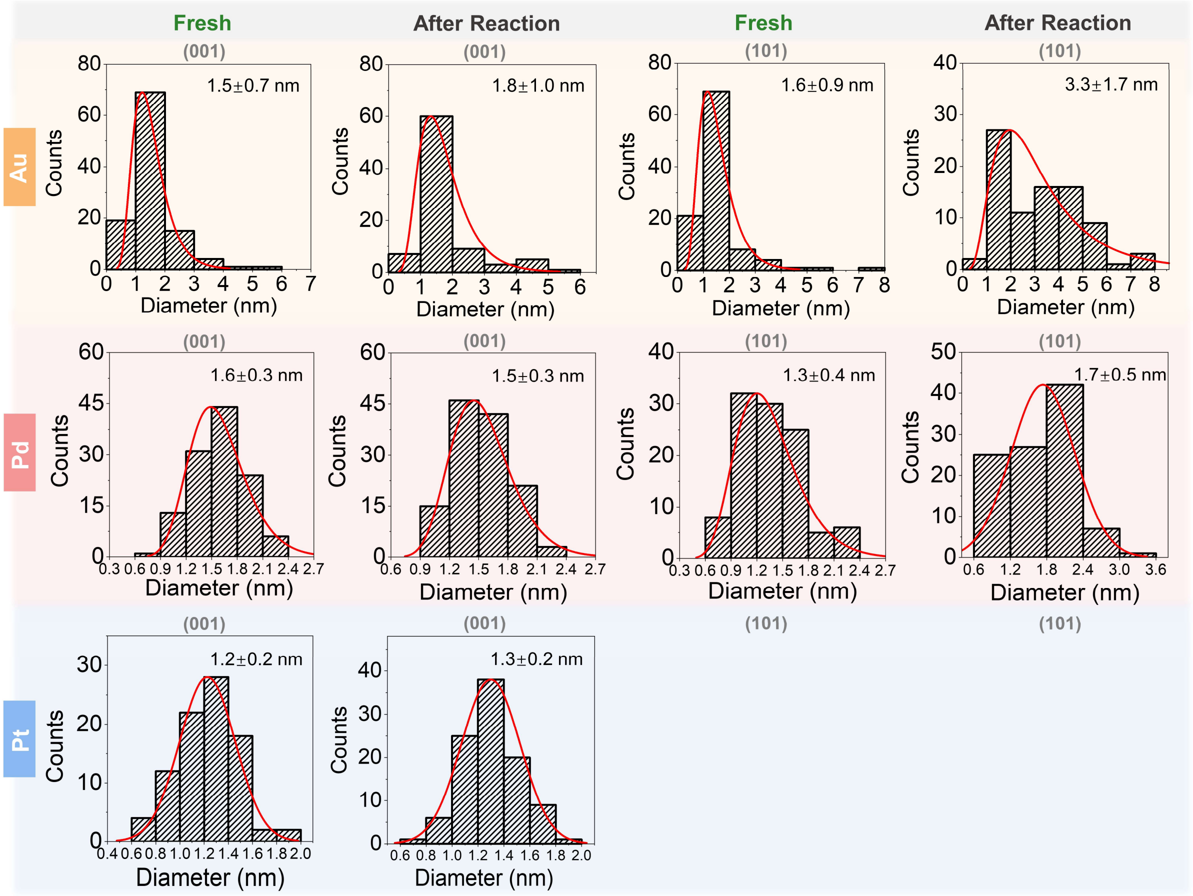

Supplementary Figure 13. Size distribution calculated from HAADF-STEM images for M-TiO_2 with {001} or {101} anatase surface (M=Au, Pd, Pt).

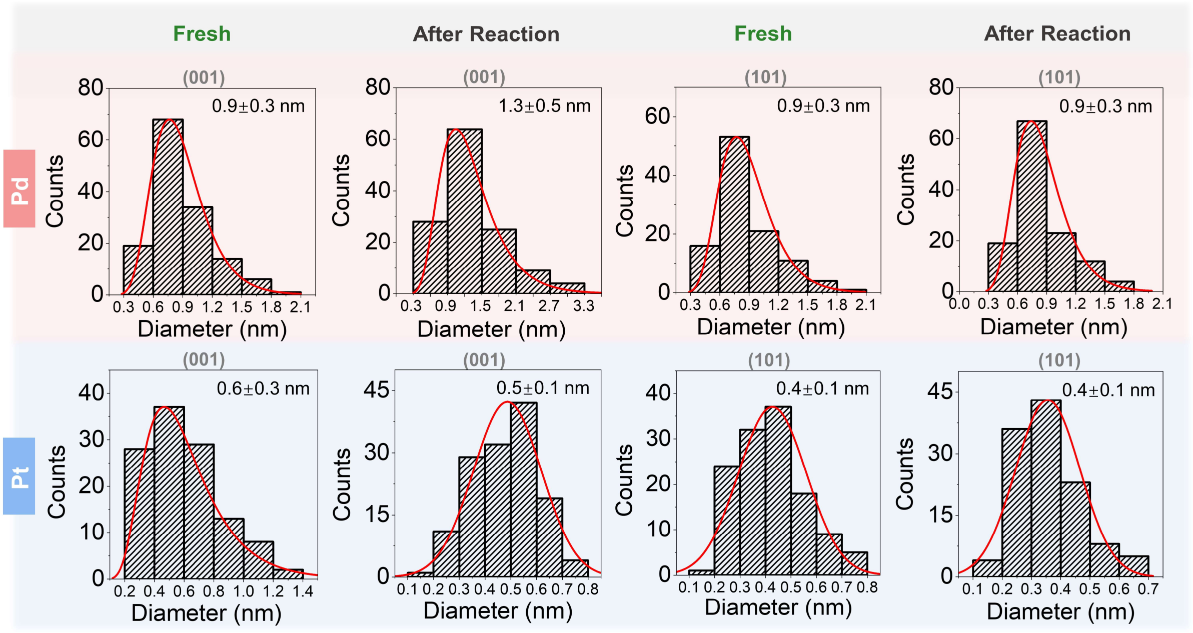

Supplementary Figure 14. Size distribution calculated from HRTEM images for M-TiO_2 with {001} or {101} anatase surface (M=Au, Pd, Pt).

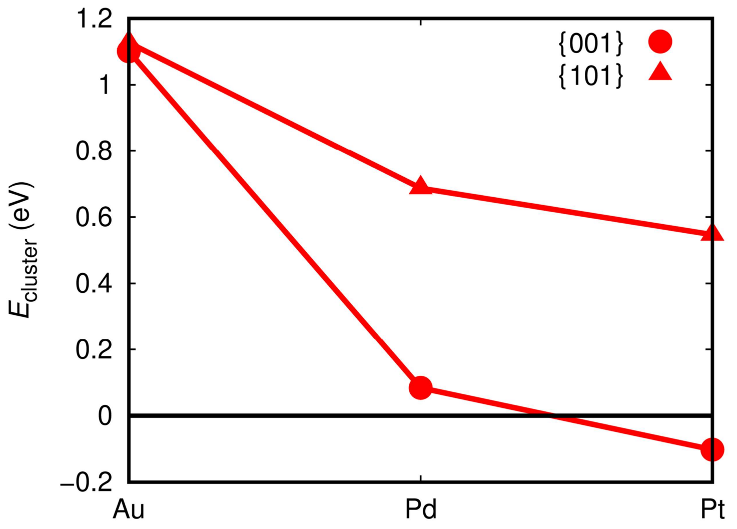

Supplementary Figure 15. DFT cluster energy results obtained for noble metal ions adsorbed on the surface of anatase TiO_2. The cluster energy is defined as $E_{cluster} = E_{ads} - E_{coh}$, where E_{ads} is the adsorption energy of the metal species and E_{coh} the corresponding cohesive energy obtained for a 9-atoms cluster. The smaller the value of the cluster energy, the smaller the tendency for metal coalescence on the TiO_2 surface.

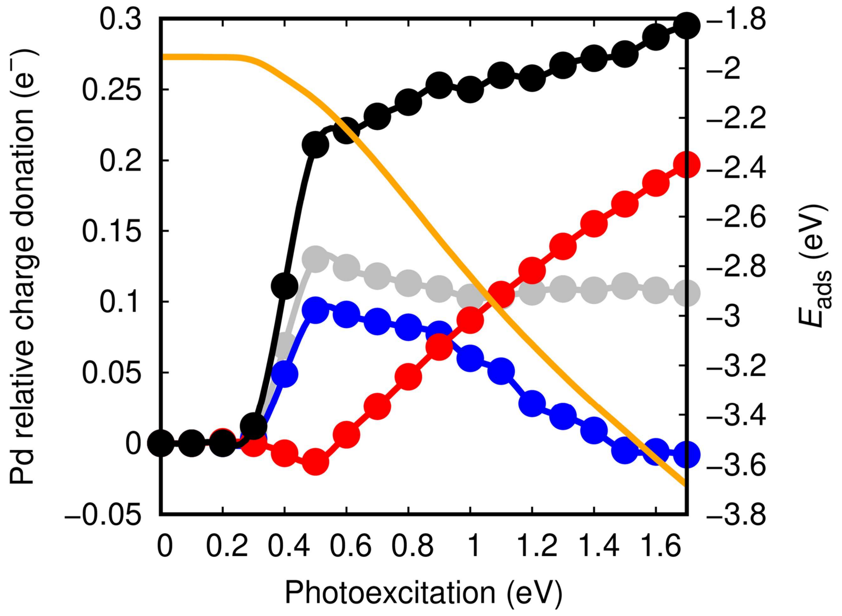

Supplementary Figure 16. Pd atoms charge donation under photoexcitation conditions on the {001} facet; DFT results are referred to non-photoexcitation conditions. The photoexcitation energy corresponds to the width of the employed electronic Fermi smearing (Methods).

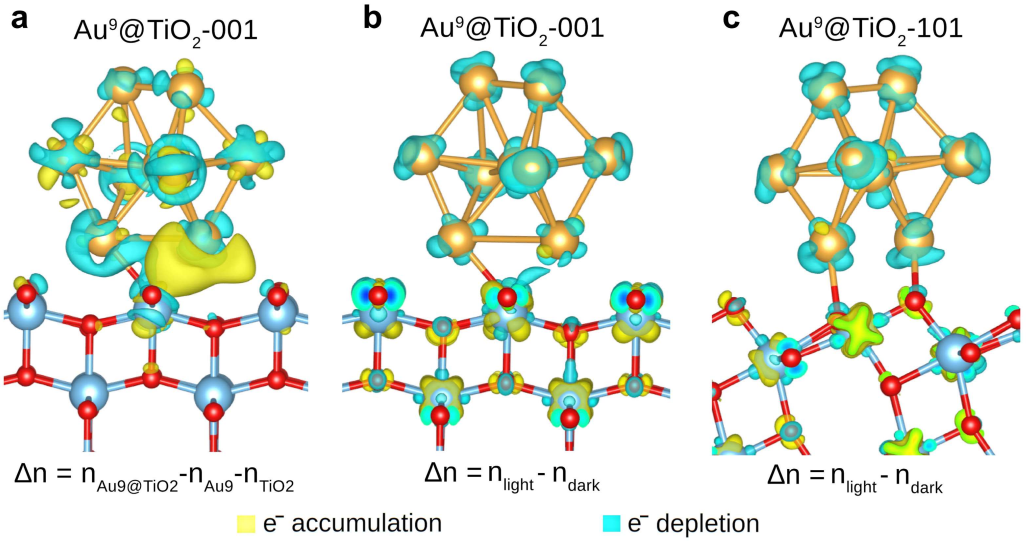

Supplementary Figure 17. Electronic charge differences calculated with first-principles DFT methods for an Au^9 nanocluster adsorbed on anatase TiO_2 surfaces. (a) Electronic charge differences with respect to the Au nanocluster and the bare TiO_2-001 surface. Atoms in the gold nanocluster present appreciable differences in their charge state as visually shown by regions of charge accumulation (yellow) and charge depletion (blue). (b-c) Electronic charge differences between photoexcitation and dark conditions for the Au nanocluster adsorbed on {001} and {101} facets.

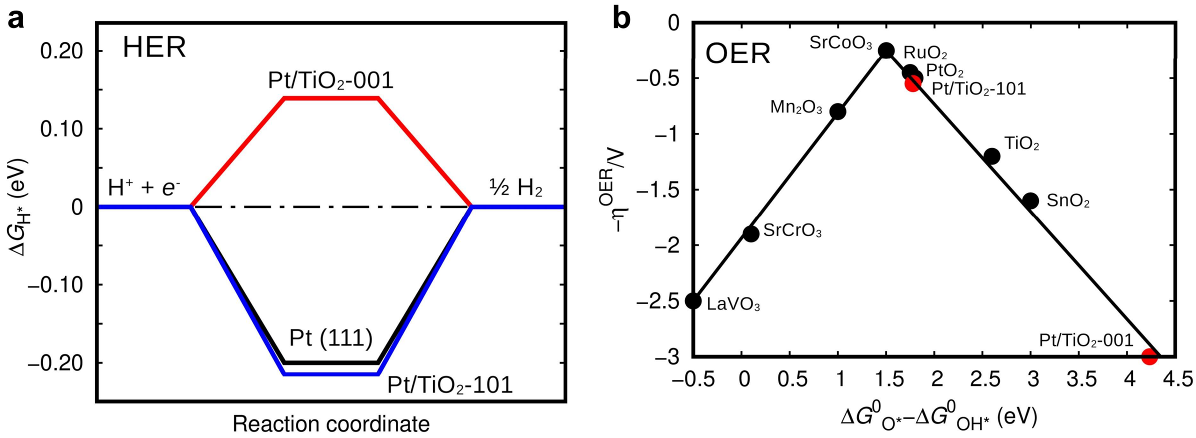

Supplementary Figure 18. First-principles density functional theory (DFT) calculations for the hydrogen evolution reaction (HER) and oxygen evolution reaction (OER) performance of anatase TiO_2-001 and TiO_2-101. HER and OER catalytic activity results are shown in (a) and (b), respectively. ΔG_X stands for the adsorption Gibbs free energy of the intermediate molecule X on the corresponding anatase TiO_2 surface. η_{OER} stands for the estimated OER overpotential. Examples of archetypal HER and OER catalysts like Pt (111), RuO_2 and $SrCoO_3$ are shown for comparison purposes.

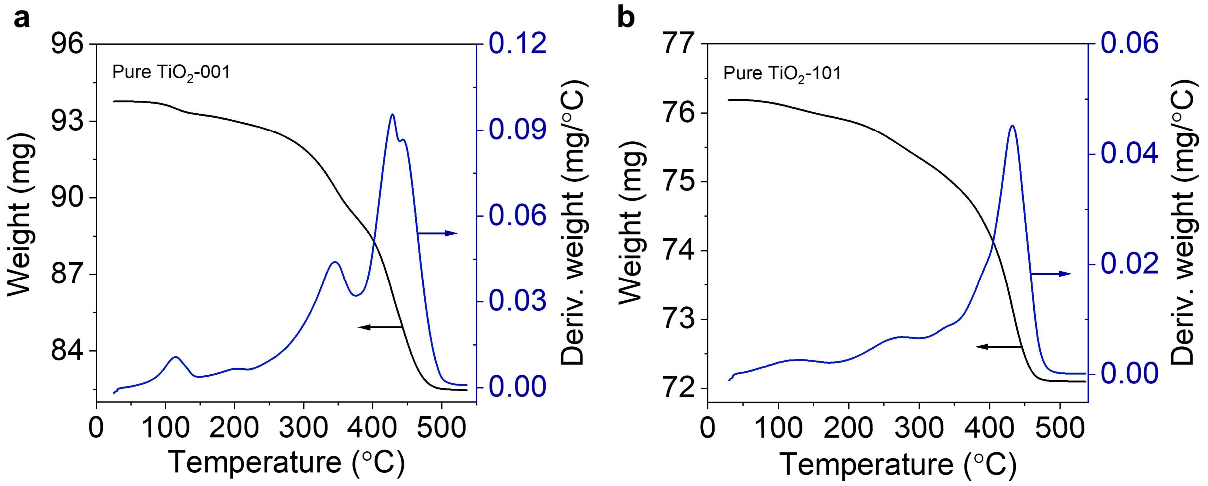

Supplementary Figure 19. TGA curves of (a): TiO_2 anatase plates and (b): TiO_2 anatase bipyramids**.**

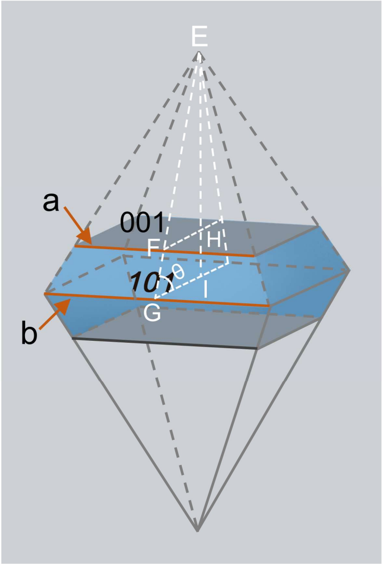

Supplementary Figure 20. Model of an anatase TiO_2 single crystal. The percentage of {001} and {101} facets has been calculated using the following equations [14–16]:

$$S_{001} = 2a^2$$

$$S_{101} = 8 \times \left(\frac{1}{2}\ EG \times b - \frac{1}{2}\ EF \times a\right)$$

$$S_{001}\% = \frac{S_{001}}{S_{001} + S_{101}} = \frac{2a^2}{2a^2 + 8 \times \left(\frac{1}{2}\ EG \times b - \frac{1}{2}\ EF \times a\right)}$$

$$EG = \frac{\frac{1}{2}b}{cos\theta} \qquad EF = \frac{\frac{1}{2}a}{cos\theta}$$

$$S_{001}\% = \frac{cos\theta}{cos\theta + \frac{b^2}{a^2} - 1}$$

$$S_{101}\% = 1 - S_{001}\%$$

θ is the theoretical value (68.3°) for the angle between anatase (001) and (101) facets, and a and b are two independent parameters of the model, as shown in the scheme.

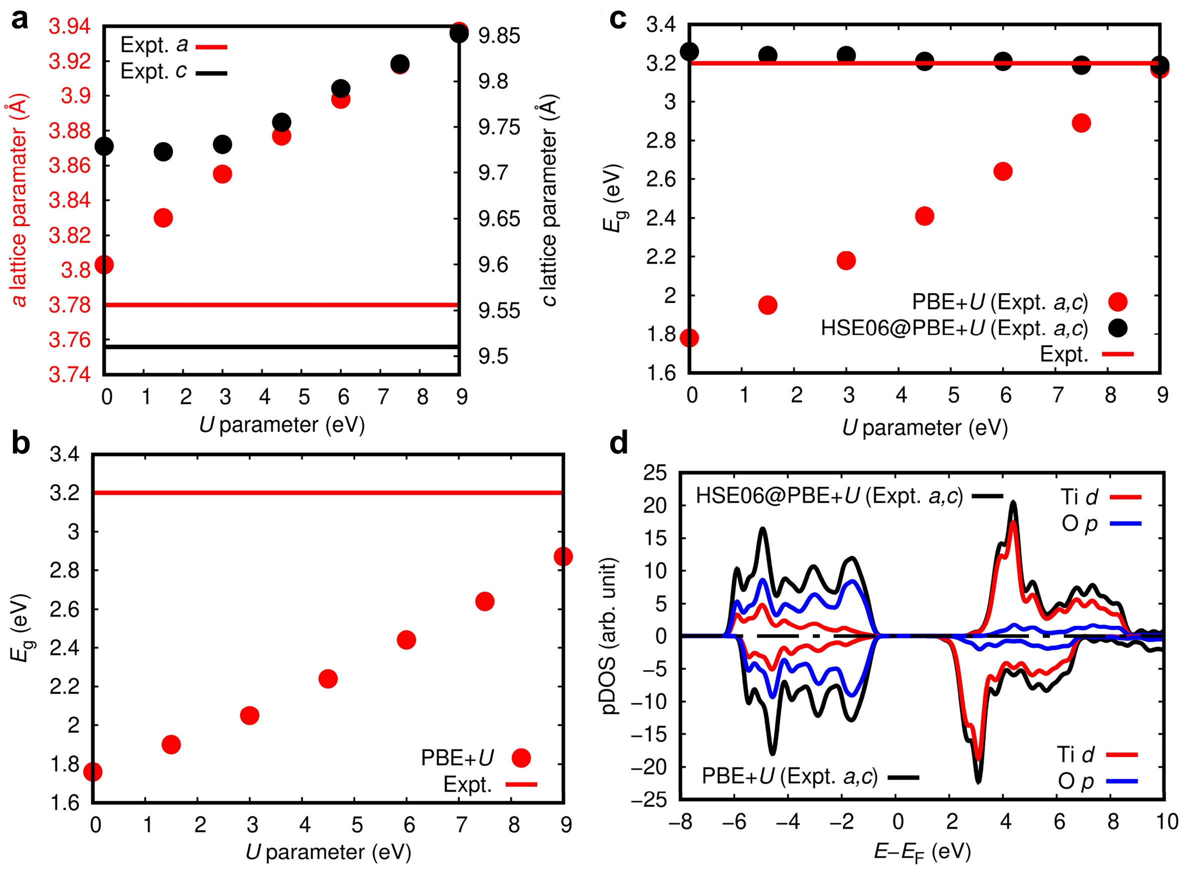

Supplementary Figure 21. Density functional theory (DFT) tests performed on the performance of the PBE+*U* method in predicting the experimental lattice vectors and band gap of anatase TiO_2. (a) Lattice vector values obtained from full PBE+U geometry relaxations. (b) Band gap values calculated with the PBE+U method on PBE+U fully relaxed structures. (c) Band gap values calculated with the PBE+U and HSE06 methods on PBE+U relaxed structures in which the value of the lattice vectors was constrained to the corresponding anatase TiO_2 experimental values. (d) Partial density of electronic states calculated for anatase TiO_2 with the PBE+U (U=3 eV) and HSE06 methods on the PBE+U (U=3 eV) relaxed structures obtained by constraining the value of the lattice vectors to the corresponding experimental values.

Supplementary Table 1. BET surface area, average pore size and total pore volume of TiO_2-001, TiO_2-101, Au/TiO_2-001 and Au/TiO_2-101 samples.

Samples	Surface area (m^2/g)	Average pore size (diameter, nm)	$^aV_{tot}$ (cm^3/g)
TiO_2-001	76.5 ± 0.2	7.7	0.21
TiO_2-101	66.5 ± 0.3	11.0	0.25
Au/TiO_2-001	55.5 ± 0.2	15.1	0.27
Au/TiO_2-101	55.9 ± 0.3	15.7	0.27

[a] Single point total pore volume at relative pressure of 0.995.

Supplementary Table 2. ICP-OES results.

Sample	Metal wt%
Au/TiO_2-001	0.91 ± 0.02
Au/TiO_2-101	0.91 ± 0.02
Pd/TiO_2-001	0.99 ± 0.04
Pd/TiO_2-101	0.92 ± 0.03
Pt/TiO_2-001	0.95 ± 0.04
Pt/TiO_2-101	0.97 ± 0.04

Supplementary References

1. Gordon, T. R. *et al.* Nonaqueous synthesis of TiO_2 nanocrystals using TiF_4 to engineer morphology, oxygen vacancy concentration, and photocatalytic activity. *J. Am. Chem. Soc.* **134**, 6751–6761 (2012).

2. Chen, Y. *et al.* Enhanced photoproduction of hydrogen on Pd/TiO_2 prepared by mechanochemistry. *Appl. Catal. B Environ.* **309**, 121275 (2022).

3. Chen, Y. *et al.* A straightforward method to prepare supported Au clusters by mechanochemistry and its application in photocatalysis. *Appl. Mater. Today* **21**, 100873 (2020).

4. Martínez, L., Soler, L., Angurell, I. & Llorca, J. Effect of TiO_2 nanoshape on the photoproduction of hydrogen from water-ethanol mixtures over Au_3Cu/TiO_2 prepared with preformed Au-Cu alloy nanoparticles. *Appl. Catal. B Environ.* **248**, 504–514 (2019).

5. Xing, C. *et al.* A Direct Z-Scheme for the Photocatalytic Hydrogen Production from a Water Ethanol Mixture on $CoTiO_3/TiO_2$ Heterostructures. *ACS Appl. Mater. Interfaces* **13**, 449–457 (2021).

6. Perdew, J. P. *et al.* Restoring the density-gradient expansion for exchange in solids and surfaces. *Phys. Rev. Lett.* **100**, 1–4 (2008).

7. Kresse, G. & Furthmüller, J. Efficient iterative schemes for ab initio total-energy calculations using a plane-wave basis set. *Phys. Rev. B - Condens. Matter Mater. Phys.* **54**, 11169–11186 (1996).

8. Dudarev, S. L., Botton, G. A., Savrasov, S. Y., Humphreys, C. J. & Sutton, A. P. Electron-energy-loss spectra and the structural stability of nickel oxide: An LSDA+U study. *Phys. Rev. B* **57**, 1505–1509 (1998).

9. Jeremy K. Burdett, Timothy Hughbanks, Gordon J. Miller, James W. Richardson Jr. & Joseph V. Smith. Structural-Electronic Relationships in Inorganic Solids: Powder Neutron Diffraction Studies of the Rutile and Anatase Polymorphs of Titanium Dioxide at 15 and 295 K. *J. Am. Chem. Soc.* **109**, 3639–3646 (1987).

10. Blöchl, P. E. Projector augmented-wave method. *Phys. Rev. B* **50**, 17953–17979 (1994).

11. Krukau, A. V., Vydrov, O. A., Izmaylov, A. F. & Scuseria, G. E. Influence of the exchange screening parameter on the performance of screened hybrid functionals. *J. Chem. Phys.* **125**, 224106 (2006).

12. Paillard, C., Torun, E., Wirtz, L., Íñiguez, J. & Bellaiche, L. Photoinduced Phase Transitions in Ferroelectrics. *Phys. Rev. Lett.* **123**, 87601 (2019).

13. Peng, B., Hu, Y., Murakami, S., Zhang, T. & Monserrat, B. Topological phonons in oxide perovskites controlled by light. *Sci. Adv.* **6**, eabd1618 (2020).

14. Yu, J., Low, J., Xiao, W., Zhou, P. & Jaroniec, M. Enhanced photocatalytic CO_2-Reduction activity of anatase TiO_2 by Coexposed {001} and {101} facets. *J. Am. Chem. Soc.* **136**, 8839–8842 (2014).

15. Yang, H. G. *et al.* Anatase TiO_2 single crystals with a large percentage of reactive facets. *Nature* **453**, 638–641 (2008).

16. Ong, W. J., Tan, L. L., Chai, S. P., Yong, S. T. & Mohamed, A. R. Highly reactive {001} facets of TiO_2-based composites: Synthesis, formation mechanism and characterization. *Nanoscale* **6**, 1946–2008 (2014).
